# Editorial: Response to societal and environmental trauma: Supporting stakeholders through health education

**DOI:** 10.3389/fpubh.2023.1160886

**Published:** 2023-03-01

**Authors:** Kimberly B. Garza, Lindsey E. Moseley, Channing R. Ford, Kristen L. Helms

**Affiliations:** ^1^Department of Health Outcomes Research and Policy, Auburn University Harrison College of Pharmacy, Auburn, AL, United States; ^2^Department of Academic Programs, Auburn University Harrison College of Pharmacy, Auburn, AL, United States; ^3^Graduate Studies, Jacksonville State University, Jacksonville, AL, United States; ^4^Alabama College of Osteopathic Medicine, Dothan, AL, United States

**Keywords:** mental health, stigma, trauma, stereotypes, resilience

Within the current political, environmental, and societal landscape, a great number of events in recent years have put a strain on mental health and led to trauma among health professionals, health educators, students, and the patients and communities they serve. This Research Topic, “*Response to societal and environmental trauma: Supporting stakeholders through health education*,” focuses on strategies and best practices for preparing current and future healthcare providers, public health educators and advocates, and members of the community to face the challenges associated with societal and environmental trauma. It presents five diverse articles that span a wide spectrum, from effects of stigma around mental health in society to how health professionals process traumatic experiences.

A large body of literature demonstrates an association between stigma toward mental illness and negative outcomes. Davis et al. assess stigma around mental health and associated medication therapy among a group of health professions students in a college of pharmacy. Student pharmacists displayed greater stigma toward use of psychotropic medications for treatment of mental health disorders than toward mental health disorders themselves. This may not be surprising given pharmacy students' focus on drug therapy within their training; however, the finding that students' level of stigma was associated with their own personal preferences for mental health treatment may impact care of patients with mental health disorders. Alternatively, Clapp et al. examined how stereotypes around mental illness are endorsed by members of the general public. They found high levels of endorsement for negative stereotypes around those who experienced trauma, including perceiving these individuals as dangerous, difficult to work with, emotionally unstable, having difficulty maintaining positive relationships, and needing formal psychological intervention in response to their trauma. Stigma surrounding mental illness can have a large impact on health and wellbeing, and these two articles demonstrate that this is still a significant problem that needs to be addressed at a broad societal level.

Other articles in this collection demonstrate the pervasiveness of poor treatment of disadvantaged populations or working conditions that lead to traumatic responses. Gurowiec et al. present an exploration of how secondary trauma resulting from helping trauma victims or those in crisis is processed among medical providers, and how this secondary trauma is related to job satisfaction. Their results demonstrate that a substantial portion of medical providers working with trauma victims develop secondary trauma, making this an important area of inquiry given the negative impacts of secondary trauma on physical, mental, and social functioning. Findings also revealed a negative association between symptoms of secondary trauma and job satisfaction, implying a possible protective role of job satisfaction on symptoms of secondary trauma. As expected, negative coping strategies were positively related to trauma-related symptoms, while positive coping strategies were negatively related. Held et al. explored healthcare provider perceptions of the lived experience of Latino communities in the Southeast United States where exclusionary policies have created anxiety and trauma in this population. They found that Latino immigrants in states with exclusionary policies experienced stressors as a result of political influences on legislation during the COVID-19 pandemic. Their work also demonstrated a high level of resilience and ethnic pride among these communities. They conclude that interventions at the micro- and macro-level, including improved access to culturally competent services and safe spaces, could improve health outcomes among Latino communities. Findings from these two articles highlight the need for interventions to mitigate risk of both primary and secondary trauma in order to improve outcomes among special populations.

The last article in this collection by Hollingsworth and Redden explores an approach to prevention of anxiety, stress, and burnout among healthcare professionals and students using an easy-to-implement intervention to support wellbeing and resiliency. Their results demonstrate a positive and sustained effect on hope and gratitude through a Tiny Habits gratitude practice that is free and simple to adopt.

Traumatic experiences have broad-reaching impact, affecting both those experiencing the trauma and those supporting the victims. Societal responses, specifically those driven by stigma and stereotypes, further exacerbate the negative effects of these experiences. Healthcare providers and health professions students can help mitigate effects of trauma by adopting and promoting approaches that improve resilience and support mental wellness. The five articles in this collection shed light on these issues and offer strategies to minimize the negative effects of trauma on mental health.

## Author contributions

KG summarized the contributed works, composed the first draft of the article, and finalized it for publication. KH, LM, and CF reviewed and revised the article critically for important content. All authors contributed to manuscript revision, read, and approved the submitted version.

